# Personalised exercise-rehabilitation for people with multiple long-term conditions (PERFORM): a randomised feasibility study

**DOI:** 10.1136/bmjopen-2025-100195

**Published:** 2025-09-17

**Authors:** Rachael A Evans, Sharon A Simpson, James R Manifield, Zahira Ahmed, Shaun Barber, Gwen Barwell, Sophie Eleanor Brown, Paulina Daw, Sarah G Dean, Patrick J Doherty, Heather Fraser, Nikki Gardiner, Colin Greaves, Tracy Ibbotson, Bhautesh Jani, Kate Jolly, Frances Mair, Emma McIntosh, Dimitrios Megaritis, Daniel Miller, Paula Ormandy, Susan Smith, Ioannis Vogiatzis, Ghazala Waheed, Tom M Withers, Rod S Taylor, Sally J Singh, on behalf of PERFORM Research Team, Sayem Ahmed

**Affiliations:** 1 Respiratory Sciences, University of Leicester, Leicester, UK; 2 Centre for Exercise and Rehabilitation Science, NIHR Leicester Biomedical Research Centre Respiratory Diseases, Leicester, UK; 3 MRC/CSO Social and Public Health Sciences Unit, University of Glasgow, Glasgow, UK; 4 Leicester Clinical Trials Unit, University of Leicester, Leicester, UK; 5 School of Sport, Exercise and Rehabilitation Sciences, University of Birmingham, Birmingham, UK; 6 Medical School, University of Exeter, Exeter, UK; 7 Health Sciences, University of York, York, UK; 8 Health Economics and Health Technology Assessment (HEHTA), University of Glasgow, Glasgow, UK; 9 University Hospitals of Leicester NHS Trust, Leicester, UK; 10 School of Health and Wellbeing, University of Glasgow, Glasgow, UK; 11 General Practice and Primary Care, School of Health and Wellbeing, University of Glasgow, Glasgow, UK; 12 Department of Applied Health Research, University of Birmingham, Birmingham, UK; 13 Department of Sport, Exercise and Rehabilitation, Northumbria University, Newcastle upon Tyne, UK; 14 University of Leicester, Leicester, UK; 15 School of Health and Society, University of Salford, Salford, UK; 16 School of Medicine, Trinity College Dublin, Dublin, Ireland; 17 Robertson Centre for Biostatistics, University of Glasgow, Glasgow, UK

**Keywords:** REHABILITATION MEDICINE, Multimorbidity, Self-Management

## Abstract

**Objective:**

Existing exercise-based rehabilitation services, such as cardiac and pulmonary rehabilitation, are traditionally commissioned around single long-term conditions (LTCs) and therefore may not meet the complex needs of adults with multiple long-term conditions (MLTCs) or multimorbidity. The aim of this study was to assess the feasibility and acceptability of the newly developed personalised exercise-rehabilitation programme for people with multiple long-term conditions (PERFORM) and the trial methods.

**Design:**

A parallel two-group mixed-methods feasibility randomised controlled trial (RCT) with embedded process and economic evaluation.

**Setting:**

Three UK sites (two acute hospital settings, one community-based healthcare setting).

**Participants:**

60 adults with MLTCs (defined as the presence of ≥2 LTCs) with at least one known to benefit from exercise therapy were randomised 2:1 to PERFORM intervention plus usual care (PERFORM group) or usual care alone (control group).

**Intervention:**

The intervention consisted of 8 weeks of supervised group-based exercise rehabilitation and structured self-care symptom-based support.

**Primary and secondary outcome measures:**

Primary feasibility outcomes included: trial recruitment (percentage of a target of 60 participants recruited within 4.5 months), retention (percentage of participants with complete EuroQol data at 3 months) and intervention adherence (percentage of intervention group attending ≥60% sessions). Other feasibility measures included completion of outcome measures at baseline (pre-randomisation), 3 months post-randomisation (including patient-reported outcomes, exercise capacity and collection of health and social care resource use) and intervention fidelity.

**Results:**

Target recruitment (40 PERFORM group, 20 control group) was met within the timeframe. Participants were 57% women with a mean (SD) age of 62 (13) years, body mass index of 30.8 (8.0) kg/m^2^ and a median of 4 LTCs (most common: diabetes (41.7%), hypertension (38.3%), asthma (36.7%) and a painful condition (35.0%)). We achieved EuroQol outcome retention of 76.7% (95% CI: 65.9% to 87.1%; 46/60 participants) and intervention adherence of 72.5% (95% CI: 56.3% to 84.4%; 29/40 participants). Data completion for attendees was over 90% for 11/18 outcome measures.

**Conclusions:**

Our findings support the feasibility and rationale for delivering the PERFORM comprehensive self-management and exercise-based rehabilitation intervention for people living with MLTCs and progression to a full multicentre RCT to formally assess clinical effectiveness and cost-effectiveness.

**Trial registration number:**

ISRCTN68786622.

**STRENGTHS AND LIMITATIONS OF THIS STUDY**

- This study evaluated the feasibility of a novel comprehensive self-management and exercise-based rehabilitation intervention specifically designed for people with a wide range of multiple long-term conditions (MLTCs) (or ‘multimorbidity’).
- The study used a mixed-methods design and included comprehensive process and economic evaluations.
- Participant selection was informed by a systematic review of exercise-based interventions for single long-term conditions.
- The MLTCs recruitment strategy was evidenced by participants with a wide range of single long-term conditions.
- There was a higher dropout rate in the control group at follow-up, as well as evidence of outcome measure burden, which we will seek to mitigate in our planned fully powered randomised trial to assess clinical effectiveness and cost-effectiveness of personalised exercise-rehabilitation programme for people with multiple long-term conditions intervention.

## Introduction

 Living with multiple long-term conditions (MLTCs; or ‘multimorbidity’), defined as two or more long-term conditions (LTCs),^1^ frequently involves complex, multiple symptoms negatively impacting daily function, health-related quality of life (HRQoL) and employment.^2 3^ MLTCs are associated with high healthcare utilisation including emergency hospital admissions and premature mortality.^4^,^6^ Population prevalence of MLTCs is increasing^7^ as a result of a number of interacting factors, including an ageing population,^8^ and changes in lifestyle, including physical inactivity^9^ and increased obesity.^10^ MLTCs therefore represent a major health and socioeconomic challenge for global healthcare systems.^11 12^

A recent overview of systematic reviews has shown that for 25 single LTCs, patients experience beneficial health outcomes from participation in exercise training.^13^ However, current exercise-based rehabilitation services, such as cardiac and pulmonary rehabilitation, commissioned on the basis of a single LTC, may not meet the complex needs of adults with MLTCs. The 2021 Cochrane review of interventions for MLTCs reported little or no improvement in patient outcomes or health system benefits such as reduced level of healthcare utilisation.^14 15^ In contrast, structured exercise-based rehabilitation appears to be a promising intervention, although previous trials in this area have been small, include a limited range of LTCs and lack a co-designed health and well-being intervention.^16^,^19^

The personalised exercise-rehabilitation for people with MLTCs (PERFORM) is a research programme funded by the National Institute of Health Research that seeks to develop and evaluate a comprehensive exercise and self-management intervention programme specifically designed for people with a wide range of MLTCs. Using a co-design approach with a range of stakeholders, including people with lived experiences of MLTCs, their caregivers, clinicians and health service commissioners, we have recently completed the development of our evidence-informed, theoretically driven PERFORM intervention programme.^20^

Following this intervention development, we undertook a mixed-methods feasibility study with the overarching aim of assessing the feasibility and acceptability of the PERFORM intervention and trial methods to inform the design of a fully powered randomised controlled trial (RCT) to assess clinical effectiveness and cost-effectiveness. The specific objectives of this study were: (1) to assess participant recruitment and retention; (2) to assess the feasibility and acceptability of the intervention exploring barriers and facilitators to uptake and engagement from both participant and healthcare provider perspectives; (3) to assess the fidelity and reach of the PERFORM intervention; (4) to further refine the PERFORM intervention and programme theory; (5) to assess the feasibility and acceptability of data collection tools; (6) to obtain estimates of key cost drivers and (7) to assess risks of bias/contamination.

This paper focuses on the quantitative element of our feasibility study, reporting our findings in terms of objectives (1), (3), (5), (6) and (7). Our companion process evaluation paper presents the methods and results of the qualitative component of the feasibility study and particularly focuses on objectives (2) and (4).^21^

## Methods

### Study design and setting

A parallel two-group feasibility trial using multiple methods was conducted to assess the feasibility and acceptability of the PERFORM intervention and the feasibility of a future fully powered RCT. The study protocol has been published previously^22^ and registered as a clinical trial. The study was conducted across three sites in the UK. Two sites were located within an acute hospital and one within a community-based health centre setting. All sites had expertise in delivering pulmonary and cardiac rehabilitation. All participants provided full written informed consent.

The study is reported in accordance with the Consolidated Standards of Reporting Trial checklist for pilot and feasibility trials.^23^

### Participants

Adults ≥18 years were considered eligible for the study if they had at least one LTC where exercise has been shown to be effective^13^ (ie, arthritis, asthma, atrial fibrillation, bronchiectasis, cancer, chronic kidney disease, chronic obstructive pulmonary disease (COPD), connective tissue disease (pain), coronary heart disease, dementia, depression, diabetes mellitus, heart failure, hypertension, long covid, multiple sclerosis, osteoporosis, painful condition, Parkinson’s disease, peripheral vascular disease, polycystic ovarian syndrome, psychoactive substance misuse, stroke or transient ischaemic attack) plus at least one other LTC from a list of 45 LTCs based on Cambridge Multimorbidity Score.^24^ Furthermore, participants had to be ambulatory (including the use of walking aids) to enable participation in the intervention and be able and willing to provide informed consent.

Participants were excluded if they were unable to give informed consent or communicate in English, had known contraindications to exercise, were unsafe to exercise without direct or continuous supervision, were unable to attend in-person sessions, had participated in an exercise rehabilitation programme in the last 6 months or had conditions compromising intervention participation, such as an unstable psychiatric disorder, being on an end-of-life pathway, an active malignancy, being on a surgical waiting list, pregnant or living in a nursing home. For people on a surgical waiting list, a pragmatic decision on inclusion was made on a case-by-case basis of the type of surgery, urgency and likely wait times.

Participants were recruited from a range of sources including outpatient clinics from relevant medical specialties in secondary care, and referrals from primary care.

### Randomisation

We aimed to recruit 60 participants to allow us to assess feasibility of the intervention and trial methods.^25^ Participants were randomly assigned in a 2:1 ratio to either the PERFORM intervention plus usual care (intervention group) or usual care only (control group). 40 participants within the intervention group allowed for at least two cohorts of PERFORM per site with 6–7 participants in each. Furthermore, the 2:1 randomisation ratio maximised recruitment to the intervention group while allowing for an understanding of feasibility under randomised conditions.

Randomisation was computer-generated and conducted using Sealed Envelope (http://www.sealedenvelope.com/) supplied by Leicester Clinical Trials Unit who were independent of the research team at sites to ensure allocation concealment.

### Interventions

#### PERFORM programme

The PERFORM intervention was developed using the Person-Based Approach.^20 26^ Details of the programme development process and content are reported elsewhere.^20^ In summary, the intervention consisted of an 8-week supervised group-based rehabilitation programme. Supervised sessions were delivered two times per week for the first 6 weeks and once a week for the final 2 weeks. These sessions lasted for 2 hours each and consisted of 1 hour of exercise training and 1 hour of health and well-being self-care support.

Exercise training was individually prescribed, progressive and involved both aerobic and resistance exercises. Aerobic exercises included walking at an intensity set at >70% peak incremental shuttle walking test (ISWT) speed.^27^ Resistance training of major muscle groups was performed in accordance with American College of Sports Medicine guidelines for older adults (three sets of 10–12 repetitions on at least two non-consecutive days per week).^28^ In addition, participants were also asked to perform exercises at home following an individually prescribed home exercise programme, aiming for 30 min bouts of exercise, 5 days per week.

Health and well-being self-care support sessions involved discussion and self-care planning, aiming to support the self-management of common symptoms and lifestyle behaviours for people with MLTCs. These sessions were developed by relevant stakeholders including individuals with MLTCs, their carers and healthcare providers, and covered a range of topics including exercise as medicine, healthy eating, improving mood and staying active. Caregivers were also welcome to attend, and written materials were supplemented to support these sessions.

In accordance with pulmonary and cardiac rehabilitation standards,^29 30^ the PERFORM intervention was delivered by healthcare professionals (eg, physiotherapist, nurse, exercise professional).

#### Usual care

Usual care involved the medical management of the MLTCs as advised by their primary/secondary care teams (ie, the continuation of medication, other treatments and medical visits). Both intervention and control groups received usual care over the course of the study.

### Outcomes

#### Feasibility outcomes

Primary feasibility outcomes were collected and assessed based on prespecified progression criteria^31^ to a full RCT (table 1). These progression criteria included: trial recruitment (percentage target of 60 randomised at end of a 4.5-month recruitment period), trial retention (percentage of randomised participants with complete EuroQol-5 dimensions-5 levels (EQ-5D-5L) data) and intervention adherence (percentage of participants allocated to PERFORM intervention attending ≥60% sessions (ie, ≥9 out of 14 sessions).

**Table 1:** Pre-specified progression criteria to a full multicentre randomised controlled trial

	Red	Amber	Green
Recruitment (% of target 60 participants met in 4.5 months following site opening)	<75%	75%–99%	100%
Retention (% of participants with complete EQ-5D data at 3-month follow-up)	<65%	65%–79%	80%–100%
Intervention adherence/attendance (% of participants attending ≥60% of sessions)	<40%	40%–59%	60%–100%

Red: do not progress to the main trial, Amber: progress if an action plan to mitigate problems can be determined and agreed with the programme steering committee, Green: progress directly to the main trial.

EQ-5D, EuroQol-5-dimensions.

Other feasibility outcomes included risk of bias/contamination (outcome assessor blinding breaks were recorded and control group participants asked at their 3-month follow-up visit if they had received any elements relevant to the PERFORM intervention). Estimates of key cost drivers were also identified (see economic evaluation section below).

#### Secondary outcomes

Secondary outcome data were collected at baseline (pre-randomisation) and 3 months post-randomisation by a blinded assessor at each study site.

Details of physical assessment tests and participant-reported outcomes (PROs) are provided in online supplemental file 1. Physical tests included the distance covered in metres during the ISWT,^32^ 4-Metre Gait Speed^33^ and hand grip strength.^34^ PROs included: HRQoL (EQ-5D-5L),^35^ mood (Patient Health-Questionnaire-9^36^ and Generalised Anxiety Disorder Assessment-7),^37^ physical activity (International Physical Activity Questionnaire),^38^ frailty (Fried Exhaustion and Weight Loss criteria),^39^ fatigue (Functional Assessment of Chronic Illness Therapy-Fatigue),^40^ pain (Brief Pain Inventory),^41^ health and disability (WHO Disability Assessment Schedule),^42^ breathlessness (Dyspnoea-12),^43^ sleep (Medical Outcome Study Sleep Scale),^44^ cognition (Montreal Cognitive Assessment),^45^ treatment burden (Multimorbidity Treatment Burden Questionnaire)^46^ and capability (ICEpop CAPability Measures for Adults (ICECAP-A)).^47^ The measurement of exercise adherence (Exercise Adherence Rating Scale)^48^ was a post-intervention measure for the PERFORM group only and compromised the blinding of the assessor. Therefore, this measurement has been omitted from any further analysis.

Clinical events (ie, mortality, hospital admissions and primary care contacts), as well as social and healthcare utilisation, medication and safety (ie, serious adverse events) were also recorded.

### Process evaluation

A process evaluation was completed following Medical Research Council guidelines for process evaluation of complex interventions^49^ and explored the feasibility and acceptability of both the intervention and study design as well as intervention fidelity. The qualitative elements of the process evaluation include interviews with patients and staff which are reported elsewhere.^21^

### Intervention fidelity

All sessions were audio-recorded and audio files scored by a member of the team who did not play a significant role in the design of the intervention (TMW). Three intervention fidelity checklists were merged to assess the quality of intervention delivery and the presence or absence of intervention components. The checklist used a Dreyfus competence-rating scale, providing a score from 0 to 5 for each item which represented the key elements of the intervention’s content and the way the designers intended the intervention to be delivered.^50^ The researcher scoring all of the files took part in a calibration exercise, where a member of the intervention design team (CG) also independently coded three initial assessment and eight health and well-being sessions and then compared scores and notes.

Examples of good practice and areas for improvement were also identified (by timestamp in the recording) and transcribed to inform future intervention training.

### Economic evaluation

Healthcare resource use data were measured in order to understand potential cost drivers in the study population and to establish the feasibility of collecting these data in a full RCT. This included health and social care contacts and number of hospital visits (both inpatient stays and outpatient appointments). Data were also collected on caregiver support and whether this was paid or unpaid.

The EQ-5D-5L and the ICECAP-A were included to assess the feasibility of measuring HRQoL and capability well-being in this study population. The EQ-5D-5L and ICECAP-A are intended to be used in a future full RCT to calculate quality adjusted life years and estimate years in full capability, respectively.^47 51 52^ EQ-5D-5L responses were mapped to EQ-5D-3L utility values for the UK population.^53 54^

### Statistical analysis

The feasibility outcomes of trial recruitment and trial retention are reported as percentages with 95% CIs. Intervention adherence is reported as the median (IQR) number of sessions attended. The proportion of participants with complete primary and secondary outcomes at 3 months for each of the proposed studies is reported overall and by study group. Mean and SD of participant secondary outcomes at baseline and follow-up by group are reported together, with mean (95% CI) baseline-follow-up change calculated along with 95% CI for each arm. Given the feasibility objectives of the study, no p values are reported.

### Patient and public involvement

The PERFORM project has an established patient advisory group (PAG) of 13 members, consisting of individuals with lived experience of MLTCs and/or participation in cardiorespiratory rehabilitation, and caregivers, representing people from deprived areas and ethnic minority groups. PAG meetings were held on Zoom approximately every 4 months, with accessible materials provided. Two PAG members attended the 6 weekly PERFORM Trial Management Group meetings to ensure close and direct communication with the wider PERFORM project team.

The PAG has shaped the PERFORM research project at every stage. For example, at their initial meeting, PAG members directed the project team to use the term ‘multiple long-term conditions (MLTCs)’ over ‘multimorbidity’ and recommended HRQoL as their preferred primary outcome. They later chose the EQ-5D-5L measure for its symptom-based co-design. The PAG has reviewed all feasibility study patient-facing documents prior to ethics submission. They also provided input on the co-design of the intervention, and the feasibility study recruitment, data collection and qualitative interviews. Recommendations included prioritising particular outcomes questionnaires to reduce participant burden and ensuring PERFORM intervention materials reflect equality and diversity considerations.^55^

## Results

### Study disposition

**Figure 1:**
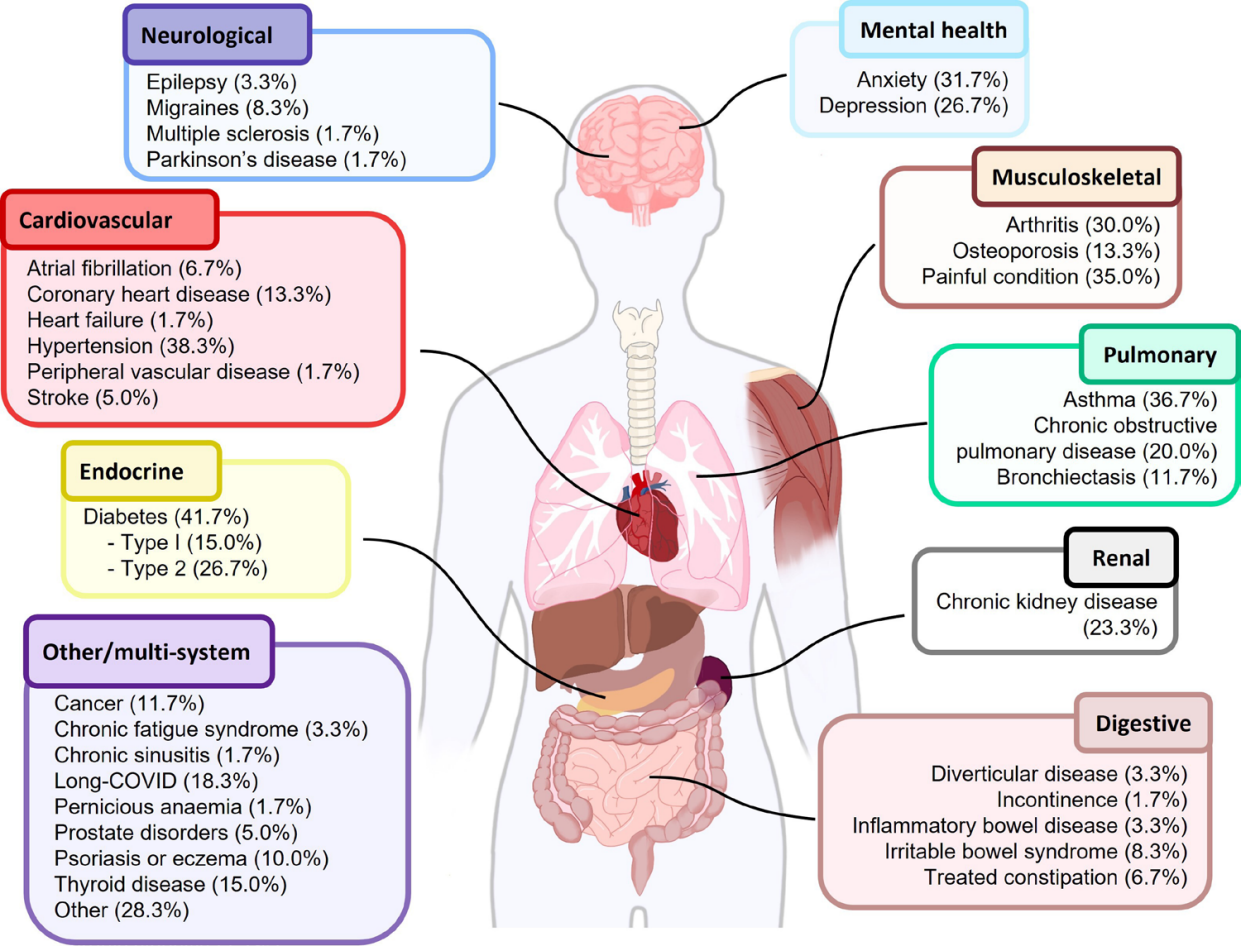
LTCs reported by participants at baseline. LTCs, long-term conditions.

A summary of individuals approached, screened, recruited and completed the study is shown in figure 1.

### Participant characteristics

The 60 recruited participants (40 PERFORM group and 20 control group) were 56.7% women, mean age 62.0 (SD 13.0) years, predominantly white British (83.3%) and with a mean body mass index (BMI) of 30.8 (SD 8.0) kg/m^2^ (see table 2). Demographic characteristics were similar between the PERFORM and control groups (table 2; online supplemental table S1). The median number of LTCs, reported by participants, was 4 (min, max: 2, 8; interquartile range: 3-6). Overall, 34 different LTCs were reported across all participants (see figure 1), with the most prevalent being: diabetes (41.7%), hypertension (38.3%), asthma (36.7%) and having a painful condition (35.0%). Reported LTCs split by treatment group are reported in online supplemental table S2. There was some evidence of differences in demographic characteristics of participants between the three sites including ethnicity (see online supplemental table S3). 50/60 participants reported their employment status, of which 60.0% (30/50) were retired, 18.0% (9/50) in full-time employment, 16.0% (8/50) of the population unable to work and 3.9% in part-time employment, with 1.9% of participants not employed (see table 2).

**Table 2:** Participant characteristics

Characteristics	All (n=60)	PERFORM group (n=40)	Control group (n=20)
Age, years (mean, SD)	62.0 (13.0)	61.4 (12.7)	63.4 (13.9)
Sex at birth, n (% female)	34 (56.7)	23 (57.5)	11 (55.0)
Ethnicity, n (%)			
White British	50 (83.3)	33 (82.5)	17 (85.0)
Asian or Asian British	6 (10.0)	4 (10.0)	2 (10.0)
Black or Black British	2 (3.3)	1 (2.5)	1 (5.0)
Mixed	2 (3.3)	2 (5.0)	0 (0.0)
BMI, kg/m^2^, mean (SD)	30.8 (8.0)	31.2 (8.1)	30.1 (7.9)
IMD scores, mean (SD)	6.0 (2.6) 6.5 (4.0–8.0)	5.5 (2.7) 6.0 (3.0–7.0)	7.2 (2.2) 8.0 (5.5–9.0)
Employment status, n (%)*			
Full-time employment	9 (15.0)	5 (12.5)	4 (20.0)
Part-time employment	2 (3.3)	2 (5.0)	0 (0.0)
Not employed but seeking	1 (1.7)	0 (0.0)	1 (5.0)
Retired	30 (50.0)	20 (50.0)	10 (50.0)
Unable to work	8 (13.3)	6 (15.0)	2 (10.0)
Missing data	10 (16.7)	7 (17.5)	3 (15.0)
Smoking status, n (%)			
Current smoker	6 (10.0)	4 (10.0)	2 (10.0)
Ex-smoker	25 (41.7)	19 (47.5)	6 (30.0)
Never smoked	27 (45.0)	16 (40.0)	11 (55.0)
Missing data	2 (3.3)	1 (2.5)	1 (5.0)
Number of LTCs, median (IQR)	4.0 (3.0–6.0)	4.0 (3.0–6.0)	4.0 (3.0–5.5)
Number of LTCs with an indication for exercise therapy, median (IQR)	3.0 (2.0–4.0)	3.0 (2.0–4.0)	3.5 (2.0–4.0)

* Some individuals answered yes to more than one employment status.

BMI, body mass index; IMD, Index of Multiple Deprivation; LTC, long-term conditions; PERFORM, personalised exercise-rehabilitation programme for people with multiple long-term conditions.

### Primary feasibility outcomes

**Figure 2:**
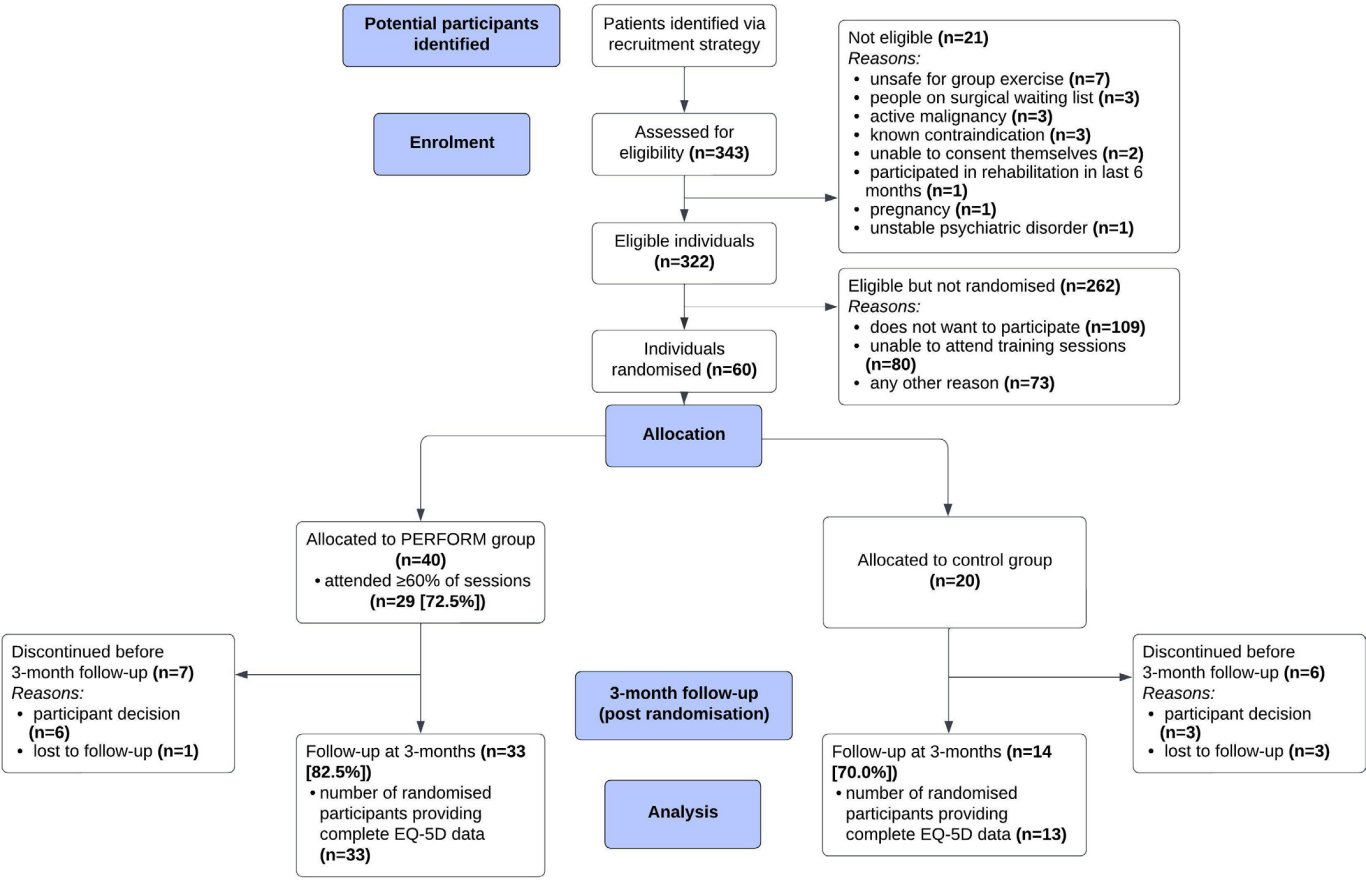
CONSORT study flow diagram. CONSORT, Consolidated Standards of Reporting Trial; EQ-5D, EuroQol-5 dimensions; PERFORM, personalised exercise-rehabilitation programme for people with multiple long-term conditions.

Recruitment took place from September 2023 to January 2024 with the final follow-up assessment conducted in April 2024. Recruitment achieved 100% of target with 60 participants recruited across three sites within the 4.5-month recruitment window (see table 3). The recruitment rate was 18.6% (60/322 eligible patients; see figure 2). 40 individuals were randomised to the PERFORM intervention group and 20 to the control group. 46 (76.7%) participants (33/40 (82.5%) PERFORM group; 13/20 (65.0%) control group) attended the 3-month follow-up assessment and had complete EQ-5D-5L data. Adherence to the PERFORM intervention was 72.5% with 29/40 participants attending ≥60% sessions (≥9/14). The median (IQR) number of sessions attended was 12 (8–14). Of those that attended the 3-month follow-up assessment, the completion rate for individual outcome measures was >90% for 11/18 outcomes (see table 4). 44.7% (21/47) of participants had a complete dataset of all outcome measures mainly reduced by incomplete Fried’s Frailty definition. Details relating to risk of bias are reported in online supplemental file 1.

**Table 3:** Feasibility primary outcome measures

Feasibility measure	Outcome
Recruitment	60 participants (100% of the recruitment target) were recruited in the 4.5-month recruitment period. (Green)
Retention	76.7% (95% CI: 65.9% to 87.1%) of randomised participants had complete EQ-5D data at 3-month follow-up. (Amber)
Intervention adherence	72.5% (95% CI: 56.3% to 84.4%) of participants in the PERFORM group attended ≥60% (≥9/14 sessions). Median of 12 sessions (IQR: 8–14) attended by participants. (Green)
Risk of bias/contamination	There were seven outcome assessor blind breaks. No participant in the control group received the PERFORM intervention.
Estimates of key cost drivers	53.3% (95% CI: 40.9% to 65.4%) of participants reported at least one instance of healthcare resource use. The highest proportion of use reported was GP visits (38.3%, 95% CI: 24.1% to 51.0%), followed by GP telephone calls, practice nurse visits and physiotherapy visits.
Feasibility and acceptability of data collection tools	44.7% of participants that attended the 3-month follow-up had complete outcome data. Qualitative process evaluation showed that some participants noted that assessments were lengthy but understood that this was necessary for the trial. Some suggested reducing the number of questionnaires.^21^
Feasibility and acceptability of intervention	Qualitative process evaluation showed that all participants interviewed found PERFORM useful and reported some positive impact.

EQ-5D, EuroQol-5-dimensions; GP, general practitioner; PERFORM, personalised exercise-rehabilitation programme for people with multiple long-term conditions.

**Table 4:** Data collection completeness of individuals that attended 3-month follow-up for proposed future primary and secondary outcome measures

	Overall (n=47) (%)	PERFORM group (n=33) (%)	Control group (n=14) (%)
EQ-5D-5L Index Score	97.9	100	92.9
EQ-5D-5L VAS	100	100	100
ISWT distance	97.9	97.0	100
4MGS	97.9	97.0	100
Hand-grip strength	97.9	97.0	100
GAD-7 score	93.6	100	78.6
PHQ-9 score	97.9	97.0	100
IPAQ-SF MET-minutes/week	83.0	87.9	71.4
FACIT-F score	89.4	87.9	92.9
BPI Pain Severity	83.0	81.8	85.7
BPI Pain Interference	85.1	81.8	92.9
WHODAS score	97.9	97.0	100
Dyspnoea-12 score	91.5	90.9	92.9
MOS-Sleep score	91.5	90.9	92.9
MoCA score	85.1	81.8	92.9
MTBQ score	91.5	90.9	92.9
ICECAP-A score	85.1	84.4	85.7
Physical frailty	61.7	63.6	57.1
All the above	**44.7**	**45.5**	**42.9**

BPI, Brief Pain Inventory; EQ-5D-5L, EuroQol-5 dimensions-5 levels; FACIT-F, Functional Assessment of Chronic Illness Therapy-Fatigue; GAD-7, Generalised Anxiety Disorder 7-Item questionnaire; ICECAP-A, ICEpop CAPability measure for Adults; IPAQ-SF, International Physical Activity Questionnaire-Short Form; ISWT, Incremental Shuttle Walk Test; MET, Metabolic Equivalents of Task; 4MGS, 4-Meter Gait Speed; MoCA, Montreal Cognitive Assessment; MOS, Medical Outcomes Study; MTBQ, Multimorbidity Treatment Burden Questionnaire; PERFORM, personalised exercise-rehabilitation programme for people with multiple long-term conditions; PHQ-9, Patient Health Questionnaire 9-Item questionnaire; VAS, Visual Analogue Scale; WHODAS, WHO Disability Assessment Schedule.

### Secondary outcomes

Results of patient-reported outcome measures and physical performance tests are presented by study group in table 5. Fried’s frailty definition and secondary ISWT outcome measure results are presented in online supplemental tables S4 and S5, respectively.

**Table 5:** Summary of secondary outcomes of exercise capacity and patient-reported outcomes at baseline and 3-month follow-up between intervention and control

	PERFORM group (n=40)			Control group (n=20)			Between-group difference of the change (95% CI)†
	Baseline	3-month	Mean change (95% CI)	Baseline	3-month	Mean change (95% CI)	
EQ-5D-5L Index Score*	0.55 (0.34)	0.60 (0.24)	0.07 (0.00 to 0.15)	0.54 (0.25)	0.55 (0.30)	0.02 (−0.05 to 0.09)	0.05 (−0.07 to 0.17)
Missing data	1	7	8	0	7	7	15
EQ-5D-5L VAS	51 (17)	58 (3)	9 (3 to 15)	55 (17)	56 (5)	6 (−8 to 8)	9 (−2 to 19)
Missing data	0	7	7	0	6	6	13
ISWT distance, m	294 (186)	334 (226)	61 (19)	404 (154)	309 (231)	−4 (32)	65 (−5 to 136)
Missing data	13	8	19	8	6	10	29
4MGS, s	4.0 (1.9)	3.8 (1.8)	−0.5 (−0.9 to –0.2)	4.0 (1.9)	4.1 (1.6)	0.0 (−0.7 to 0.6)	−0.5 (−1.2 to 0.2)
Missing data	7	8	14	3	6	7	21
Hand-grip strength Dominant hand, kg	29.3 (10.8)	32.1 (10.6)	2.6 (0.7 to 4.5)	29.2 (9.0)	25.7 (12.2)	−0.8 (−5.5 to 4.0)	3.4 (−0.7 to 7.4)
Missing data	2	8	10	1	6	7	17
GAD-7 score	6.4 (1.1	4.8 (0.9)	−1.4 (−2.8 to 0.0)	5.4 (1.2)	5.0 (1.6)	−0.5 (−3.4 to 2.5)	−0.9 (−3.7 to 1.9)
Missing data	3	7	10	1	9	9	19
PHQ-9 score	8.9 (7.0)	7.7 (6.1)	−1.1 (−2.6 to 0.4)	8.7 (6.7)	8.6 (5.4)	−0.6 (−4.0 to 2.8)	−0.5 (−3.5 to 2.5)
Missing data	1	8	9	0	6	6	15
IPAQ-SF MET-minutes/week	2258 (2742)	2168 (1446)	−81 (−1249 to 1088)	2364 (1882)	2090 (2557)	−437 (−2843 to 1969)	357 (−1973 to 2686)
Missing data	12	11	16	6	10	12	28
FACIT-F score	27.9 (1.9)	32.3 (2.2)	3.6 (1.1 to 6.0)	26.2 (2.9)	25.3 (3.9)	−0.2 (−5.1 to 4.8)	3.7 (−1.0 to 8.4)
Missing data	5	11	13	1	7	7	20
BPI Pain Severity	5.0 (1.7)	4.4 (2.1)	0.1 (−0.4 to 0.6)	5.0 (1.6)	4.6 (2.5)	0.3 (−0.3 to 0.9)	−0.2 (−1.0 to 0.6)
Missing data	15	13	20	4	8	10	30
BPI Pain Interference	4.8 (2.5)	4.1 (2.5)	0.3 (−0.6 to 1.2)	4.8 (2.3)	3.9 (3.1)	0.2 (−1.0 to 1.4)	0.1 (−1.3 to 1.5)
Missing data	16	13	21	4	7	9	30
WHODAS score	23.5 (19.4)	25.3 (19.9)	1.7 (−1.7 to 5.1)	22.5 (15.3)	27.8 (21.8)	3.6 (−3.6 to 10.9)	−1.9 (−8.7 to 4.8)
Missing data	1	8	9	1	6	7	16
Dyspnoea-12 score	8.5 (1.5)	7.0 (1.5)	−0.7 (−2.7 to 1.4)	8.5 (2.1)	9.5 (2.5)	1.3 (−1.8 to 4.3)	−2.0 (−5.5 to 1.5)
Missing data	6	10	13	1	7	7	20
MOS score	37.7 (24.3)	35.5 (22.2)	−0.5 (−5.5 to 4.5)	49.6 (25.8)	49.0 (24.2)	−4.1 (−13.1 to 4.9)	3.6 (−5.6 to 12.8)
Missing data	5	10	12	1	7	7	19
MoCA score	24.6 (2.6)	25.4 (2.9)	0.5 (−0.3 to 1.3)	25.6 (2.5)	27.2 (1.8)	0.4 (−0.9 to 1.7)	0.1 (−1.3 to 1.5)
Missing data	1	13	13	0	7	7	20
MTBQ score	18.1 (21.8)	14.8 (16.8)	−2.3 (−6.7 to 2.2)	15.6 (15.9)	22.3 (21.3)	6.3 (0.0 to 12.6)	−8.5 (−16.0 to 1.0)
Missing data	6	10	13	1	7	7	20
ICECAP-A score	0.76 (0.23)	0.78 (0.19)	0.02 (−0.02 to 0.07)	0.79 (0.16)	0.69 (0.24)	−0.11 (−0.21 to –0.01)	0.14 (0.05 to 0.23)
Missing data	7	12	15	1	8	8	23

Data are presented as mean (SD) or mean change (95% CI).

* Proposed primary outcome measure for the future trial.

† PERFORM minus control.

BPI, Brief Pain Inventory; EQ-5D-5L, EuroQol-5 dimensions-5 levels; FACIT-F, Functional Assessment of Chronic Illness Therapy-Fatigue; GAD-7, Generalised Anxiety Disorder 7-Item questionnaire; ICECAP-A, ICEpop CAPability measure for Adults; IPAQ-SF, International Physical Activity Questionnaire-Short Form; ISWT, Incremental Shuttle Walk Test; MET, Metabolic Equivalents of Task; 4MGS, 4-Metre Gait Speed; MoCA, Montreal Cognitive Assessment; MOS, Medical Outcomes Study; MTBQ, Multimorbidity Treatment Burden Questionnaire; PERFORM, personalised exercise-rehabilitation programme for people with multiple long-term conditions; PHQ-9, Patient Health Questionnaire 9-Item questionnaire; VAS, Visual Analogue Scale; WHODAS, WHO Disability Assessment Schedule.

No serious adverse events were reported during the period of the study.

### Intervention fidelity

15 initial assessments, 51 health and well-being sessions and 2 maintenance sessions were included in the analysis. Several recordings had poor audibility or cut-off before the end of the session, so were only partially scored. Scores could range from 5 (very good evidence of fidelity) to 0 with a score of ≥3.0 representing competent delivery. The mean (SD) overall scores for initial assessment, health and well-being and maintenance sessions were 2.4 (0.7), 1.9 (0.6) and 2.9 (0.6), respectively. Where a score of 3 represents competent delivery. The individual item scores are presented in online supplemental table S6.

The highest mean scoring items were assessing needs and concerns of patients 3.3 (0.9), ensuring a focused discussion 3.7 (1.0) and reflective listening 4.5 (0.7) for the initial assessment, health and well-being and maintenance sessions, respectively. Engaging social support was the lowest scoring item for all session types with a mean of 0.3 (0.6), 0.8 (1.0) and 1.5 (0.7) for initial assessment, health and well-being and maintenance sessions, respectively.

Examples of good practice in the recordings include the use of reflective listening and open-ended questions. There were also numerous examples of delivery aspects that could be improved. These included skipping action-planning and progress-review sections, and over-sharing of personal experiences. Qualitative data relating to intervention fidelity are reported in our companion paper.^21^

### Economic evaluation

10 different categories were captured in the collection of healthcare resource use data at 3 months. General practitioner (GP) visits were the most frequently used healthcare resource, followed by GP telephone calls and practice nurse visits (see online supplemental figure S1).

Mean (SD) EQ-5D-5L index scores of 0.55 (0.34) and 0.60 (0.24) were seen for the PERFORM group at baseline and 3 months follow-up, respectively (see table 5). Mean (SD) utility values for the control group were 0.54 (0.25) and 0.55 (0.30) at baseline and 3 months, respectively. The ‘pain’ and ‘usual activities’ domains had the highest number of participants reporting moderate, severe or extreme problems, while the ‘Self Care’ domain had the highest number of participants reporting ‘no problems’ (see online supplemental figure S2).

Mean (SD) capability well-being values derived from ICECAP-A responses in the intervention group were 0.76 (0.23) and 0.78 (0.19) at baseline and 3 months follow-up, respectively, while in the control group, mean capability values were 0.79 (0.16) and 0.69 (0.24) at baseline and 3 months, respectively (see table 5). The domain of ‘feeling settled and secure’ had the highest number of participants reporting no capability, while the domain of ‘Love, friendship and support’ had the highest number of participants reporting full capability (see online supplemental figure S3).

## Discussion

We report the feasibility of a novel comprehensive exercise-based rehabilitation intervention alongside symptom-focused health and well-being sessions for adults living with a wide range of MLTCs. Our study achieved the pre-specified criteria for progression to full RCT for both participant recruitment and intervention adherence to the intervention. While indicative of progression, the third criteria of retention rate (completion of EQ-5D data at 3 months follow-up) indicated that improvements in outcome completion would be needed for a future full RCT. While overall participant completion rates of individual outcomes were generally good, there was evidence of participant outcome burden (<50% of participants providing complete data at both baseline and follow-up assessments).

This study sought to specifically target people with a wide range of MLTCs. Study participants had a median of four LTCs that included over 35 different conditions involving at least nine different bodily systems. These findings are supportive of the eligibility criteria and recruitment strategies employed across the three sites in this feasibility study. While patients with an index indication meeting referral to a cardiac or pulmonary rehabilitation (including coronary artery disease, heart failure, COPD and pulmonary fibrosis) were not excluded from this feasibility study, the majority of study participants did not have cardiorespiratory disease, thus reflecting the novelty of our study population. Our recruitment strategy was designed with patients and clinicians, with a clear direction from PAG members reporting that they preferred the referral for rehabilitation and the PERFORM study to be generated by their specialist team rather than their primary care team. We actively approached a variety of clinical specialties (eg, chronic kidney disease and diabetes clinics) across our three study sites to ensure a wide range of LTCs were included. In the future trial, we would replicate this strategy. We would, however, refine our eligibility criteria as a small number of participants (n=2) had minimal deficit in physical function (ISWT >80% predicted).^56^ For a future trial, we would define an upper level of exercise capacity as an exclusion criteria ceiling of function or fitness as entry criteria.

Interventions to date for MLTCs have centred around self-management support, pharmacological management including deprescribing and care coordination.^14^ The 2021 Cochrane systematic review of 16 randomised trials in over 4700 people with MLTCs did not show meaningful improvements in any of the assessed interventions in terms of HRQoL or other health-related outcomes. While not powered to demonstrate statistical superiority in secondary outcomes, the results of this feasibility study are promising with a consistent direction of improvement across all patient-reported and exercise outcomes in the PERFORM intervention group compared with control.

These positive directions in secondary outcomes are consistent with those reported in previous evaluations of exercise-based rehabilitation interventions for people with MLTCs.^17^,^19^ However, in contrast to previous trials, our PERFORM research programme introduces two fundamental innovations to better reflect real-world management of MLTCs. First, we seek to take a ‘disease agnostic’ approach to MLTCs definition and patient selection by including a wide range of LTCs as possible (provided that participants report at least one LTC with evidence supporting exercise training).^13^ Second, our PERFORM intervention has been designed by combining intervention elements of both exercise training alongside self-management sessions in order to maximise the promotion of MLTCs participant health and well-being.

As the benefits of exercise therapy are increasingly realised for a wide range of LTCs, it becomes increasingly untenable to design and implement single LTC or disease-specific exercise-based rehabilitation programmes. The current PERFORM intervention was uniquely designed with patients and clinicians for adults with MLTCs using a symptom-focused approach with a health and well-being education programme designed to support effective symptom management and positive lifestyle behaviours to enhance HRQoL. Previous work has shown the benefit of combining rehabilitation for cardiac and pulmonary diseases^57^ including capacity development of the intervention delivery teams.^58^ The PERFORM intervention has therefore been designed to be delivered by a wide range of healthcare professionals from a variety of disciplines.^13^ Rehabilitation needs to be acceptable and accessible for successful implementation in the future. Importantly, we also involved a community site and included delivery by a range of healthcare and exercise professionals.

Some 40% of participants in the present study (20/50) were either in some form of employment, seeking employment or unable to work due to ill health and 48% (29/60) were of working age. This emphasises the broader socioeconomic impacts of MLTCs and highlights the need for interventions to improve not only health outcomes but also promote return to work, where applicable. Furthermore, our baseline PROs reflect the degree of burden experienced by the MLTCs population recruited in this feasibility study. The ICECAP-A scores seen in the study are in line with reported scores 0.45–0.83 for people with moderate or severe arthritis, asthma, cancer, depression, diabetes or heart disease, and lower than healthy UK population estimates (0.88, SD: 0.14).^59^ Our EQ-5D-5L scores of 0.54–0.60 indicate lower levels of HRQoL than those typically seen in single LTC rehabilitation populations, such as COPD (0.70),^60^ and the general population (0.66–0.93).^61^

While we met our three pre-specified progression criteria to progress to a larger definitive RCT, the finding of this feasibility study and companion process evaluation^21^ informed a number of proposed adaptations for the design for a future fully powered RCT (see box 1).

Box 1 **Proposed adaptations to the design of a future full PERFORM randomised trial of PERFORM intervention**

- *Retention*: (1) Adopt evidence-based procedures to try to reduce loss to follow-up noting our higher dropout rate in the control group. We will be formally testing, as part of a SWAT, the use of a Facebook page for trial participants to improve engagement throughout which will be conducted as a trial study within a trial.
- *Burden of outcome measures*: (2) The Montreal Cognitive Assessment and the WHODAS will not be used in a future trial. (3) Participants will be offered the opportunity to complete PRO questionnaires ahead of their scheduled research visit to reduce burden and for researchers to check completion at the visit. (4) If a participant is unable to return for a scheduled visit, the PROs will be attempted to be collected by telephone and, as a minimum, the primary outcome measure (EQ-5D-5L).
- *Participant selection*: (5) Specify exclusion of an upper limit of physical function and exercise capacity to identify a rehabilitation need. (6) Eligibility to include one LTC from the list of 25 LTCs with an indication for exercise-based rehabilitation, and any other LTCs. (7) Maintain PPI to ensure subsequent recruitment remains inclusive and representative.
- *Outcome measure collected*: (8) As 50% of participants were working age, we will be using the work productivity and activity impairment questionnaire in the future trial to potentially inform broader societal economic impacts of the intervention. (9) Based on inpatient hospital data collected in the feasibility study, the data collection instruments have been amended to ensure that number of nights spent in hospital is recorded. (10) The endurance shuttle walk test will be added as it is already performed for the exercise prescription and is a sensitive outcome measure.
- *Blinding*: (11) The Exercise Adherence Rating Scale will not be used in a future trial as it was intended as a 3-month assessment for the PERFORM group only and compromised assessor blinding. (12) Ensure preparation of participants to not share group allocation during follow-up assessment.

EQ-5D-5L, EuroQol-5 dimensions-5 levels; LTCs, long-term conditions; PERFORM, personalised exercise-rehabilitation programme for people with multiple long-term conditions; PPI, patient and public involvement; PRO, participant-reported outcome; SWAT, study within a trial; WHODAS, WHO Disability Assessment Schedule.

### Strengths and limitations

Two key strengths of this study were the comprehensive nature of the PERFORM intervention and our broad approach to MLTCs recruitment. In contrast to previous exercise intervention trials that have focused on a limited group of LTCs (eg, musculoskeletal plus a cardiac condition or diabetes plus a mental health condition),^19^ we have designed our PERFORM programme to be suitable for an MLTC population irrespective of their individual combination LTC. This was reflected in our co-developed PERFORM intervention that provides a comprehensive rehabilitation programme that combines the principles of self-management education and exercise training. Furthermore, we specifically designed our recruitment strategy to reflect the ‘real-world’ of MLTCs by allowing people with effectively any combination of two or more LTCs to participate. The only restriction to study entry was that participants had to have at least one LTC shown to benefit from exercise intervention.^13^ Other strengths of this feasibility trial included the inclusion of a rigorous process evaluation with assessment and refinement of the programme theory, an economic evaluation and recruitment of a diverse group of participants including a wide range of LTCs and demographics comparable with national statistics including ethnicity^62^ and socioeconomic deprivation within an MLTC population.^63^ In the future trial, we will endeavour to ensure wide geographical coverage from inner city to rural and coastal locations.

A limitation of the current intervention is the ‘centre-based’ modality which for some patients will limit accessibility, for example, due to transport, timing when working and group-based activities for people with particular mental health impairments. However, there are a number of reasons for this choice: (1) this is a novel population being investigated; patient safety is key and requires supervision at this stage in intervention development and testing; (2) there is not clear equivalence in effectiveness for centre-based or digital remote interventions for exercise-based interventions and (3) for some patients, the peer support is an essential part of the intervention.^30 64^ A further limitation is the short-term nature of the intervention and follow-up. However, this is specifically due to the unclear results of formal maintenance programmes even for populations such as COPD where short-term programmes have been implemented for over three decades.^65^ Short-term follow-up was pragmatic for resourcing a feasibility study. The relatively small sample size and level of data completeness within this feasibility trial should be noted. Our future multicentre RCT aims to be fully powered to a priori detect significant changes in the primary outcome (EQ-5D-5L).

## Conclusions

Our findings support the feasibility and rationale for delivering the PERFORM comprehensive self-management and exercise-based rehabilitation intervention for people living with MLTCs and progression to a full multicentre RCT to formally assess clinical effectiveness and cost-effectiveness.

## Supplementary material

10.1136/bmjopen-2025-100195 online supplemental file 1

## Data Availability

Data are available upon reasonable request.
