# Supplementary material for: Personalised exercise-rehabilitation for people with multiple long-term conditions (PERFORM): a randomised feasibility study
Source: BMJ Open. 2025 Sep 17;15(9):e100195. doi: 10.1136/bmjopen-2025-100195 (PMC12458827; doi:10.1136/bmjopen-2025-100195)
Supplement: online supplemental file 1 [file bmjopen-15-9-s001.docx]

**Supplementary material**

**Physical assessment tests**

***Exercise capacity***

The incremental shuttle walk test (ISWT) was used as a measure of maximal exercise capacity. This test is a progressive, maximal test where participants walked 10-metre shuttles at a speed externally paced by an audio tape, as described previously.^1^ They were instructed to reach (<0.5m away) the opposite cone at the time of the “bleep”. These bleeps increased in frequency every minute which was signalled by a triple bleep, indicating to the participant to increase their walking speed by 0.17 m/s. Participants were required to walk until they were unable to keep up with the walking pace. The test was terminated if the participant was >0.5 m away from the cone during the bleep on two consecutive occasions. No encouragement was provided by outcome assessors during the test.

The ISWT was performed twice at baseline with the first being a practice test to familiarise each participant with the procedures. The second “true” test was performed following sufficient recovery (at least 30 minutes after the practice test). The main outcome from the ISWT is distance covered in metres (representing completed shuttles only). Secondary outcomes of heart rate, oxygen saturation (SpO_2_), Borg breathlessness, and ratings of perceived exertion (RPE) were recorded following the test. Furthermore, reasons for termination were recorded, including: shortness of breath, leg fatigue, pain, or other.

***Gait speed***

The time taken in seconds for participants to walk 4 metres at usual walking speed (Four-metre gait speed [4MGS] was assessed along a flat, unobstructed course.^2^

***Strength***

Maximum hand grip strength^3^ was measured using a hand-held dynamometer and performed three times on both the dominant and non-dominant hand. The highest score was taken per hand and reported in the results for the dominant hand only.

**Participant-reported outcomes**

***Health-related quality of life***

The European Quality of Life 5-Dimensions 5 Levels (EQ-5D-5L) questionnaire was used as a measure of health-related quality of life.^4^ This questionnaire defines health in terms of mobility, self-care, usual activities, pain or discomfort, and anxiety or depression. The EQ-5D visual analogue scale (VAS) records the patient’s self-rated health on a visual scale that ranges from ‘the best health you can imagine’ to ‘the worst health you can imagine’. The EQ-5D-5L is a valid measure and responsive to change following pulmonary rehabilitation with a minimal clinically important difference (MCID) of 0.05 (utility index) and 7.0 (VAS).^5^

***Depression***

The patient health questionnaire (PHQ-9)^6^ was used to assess the presence and/or severity of depression. The measure includes 9 items scored between 0 (not at all) to 3 (nearly every day), with higher scores indicating increased depression severity.

***Anxiety***

The Generalised Anxiety Disorder Assessment (GAD-7)^7^ was used to assess the presence and/or severity of anxiety. The measure comprises of 7 items that are scored between 0 (not at all) and 3 (nearly every day), with higher scores representing a greater level of anxiety.

***Fatigue***

The Functional Assessment of Chronic Illness Therapy – Fatigue (FACIT-F)^8^ was used to assess fatigue. This measure is a 13-item questionnaire that assesses self-reported fatigue. Participants respond to each item using a 4-point Likert scale ranging from 0 (not at all) to 4 (very much). After the negatively stated items are reversed, the total score was calculated with higher scores indicating greater fatigue.

***Pain***

The Brief Pain Inventory (BPI)^9^ was used to measure the intensity of pain and degree of pain relief provided by medications. The BPI also measures interference of pain in the individual’s life, including the degree that pain interferes with general activity, walking, work, mood, relations with others and sleep.

***Breathlessness***

The dyspnoea 12 questionnaire^10^ was used to assess breathlessness, and includes 12 items that are scored between 0-3. A total score is then calculated that provides a global score of breathlessness severity that includes both physical and affective elements. Higher scores represent greater sensations of breathlessness.

***Physical activity***

The international physical activity questionnaire (IPAQ)^11^ is a self-reported measure for physical activity. Participants were asked to recall their physical activity from the past 7 days. All Walking, Moderate and Vigorous time variables exceeding ‘3 hours’ or ‘180 minutes’ was be truncated (re-coded) to be equal to ‘180 minutes’ in a new variable to allow a maximum of 21 hours of activity in a week to be reported for each category (3 hours * 7 days).

***Sleep***

The Medical Outcome Study Sleep Scale (MOS Sleep)^12^ is a 12-item questionnaire that assesses sleep disturbance, sleep adequacy, somnolence, quantity of sleep, snoring, and awakening short of breath or with a headache.

***Treatment burden***

The Multi-morbidity Treatment Burden Questionnaire (MTBQ)^13^ is a 10-item questionnaire that aims to measure treatment burden in patients with multi-morbidity.

***Frailty***

The Fried frailty criteria^14^ was utilised in this trial based on the presence of slowness, weakness, self-reported exhaustion, unintentional weight loss, and low physical activity, whereby participants were classified as ‘robust’ (0 criteria present), ‘intermediate’ (1 or 2 criteria present), or ‘frail’ (≥3 criteria present).

Criteria for slowness was assessed using 4MGS. For males, slowness was defined as ≥6.12 seconds (height ≤173cm) or ≥5.25 seconds (height >173cm). For females, this was ≥6.12 seconds (height ≤159cm) or ≥5.25 seconds (height >159cm).

Weakness was assessed by dominant handgrip strength (DHS). For males, weakness was present depending on the following DHS and body mass index (BMI) cutofffs. BMI: ≤24 and DHS: ≤29, BMI: 24-28 and DHS: ≤30, or BMI: >28 and DHS: ≤32. For females, weakness was present if: BMI: ≤23 and DHS: ≤17, BMI: 23-26 and DHS: ≤17.3, BMI: 26-29 and DHS: ≤18, or BMI: >29 and DHS: ≤21.

Self-reported exhaustion was assessed using 2 questions from the Center for Epidemiologic Studies Depression (CES-D) scale.^15^ Participants scored between 0 (rarely or none of the time: <1 day), 1 (some or a little of the time: 1–2 days), 2 (a moderate amount of the time: 3–4 days) and 3 (most of the time), with higher scores indicating increased depression severity. Subjects answering “2” or “3” to either of these questions were categorised as frail by the exhaustion criterion.

Unintentional weight loss was assessed as self-reported unintentional weight loss in the last year. For this, participants were asked if they have unintentionally lost more than 4.5kg in the last 12 months.

Low physical activity was assessed using the IPAQ if the physical activity is not categorised as moderate or vigorous. Moderate physical activity = 3 or more days of vigorous-intensity activity of at least 20 minutes per day OR 5 or more days of moderate-intensity activity and/or walking of at least 30 minutes per day OR 5 or more days of any combination of walking, moderate-intensity or vigorous intensity activities achieving a minimum total physical activity of at least 600 MET-minutes/week. Vigorous physical activity = vigorous-intensity activity on at least 3 days (20min minimum, achieving a minimum total physical activity of at least 1500 MET-minutes/week OR 7 or more days of any combination of walking, moderate-intensity or vigorous-intensity activities achieving a minimum total physical activity of at least 3000 MET-minutes/week.

***Health and disability***

The World Health Organisation Disability Assessment Schedule 2.0 (WHODAS 2.0)^16^ is a 36-item measure that assesses disability in adults age 18 years and older. The total score was calculated, whereby the sum of the 36-items scored 0–4 ranges from 0 to 144. The resulting score will be transformed into a 0–100 scale, with higher numbers indicating a higher level of disability.

***Cognition***

The Montreal Cognitive Assessment (MoCA)^17^ was used to assess cognition. This questionnaire is a 30-point cognitive screening test designed to help detect mild cognitive impairment and Alzheimer's disease. It includes items that assess short-term memory, visuospatial abilities, orientation, executive function, concentration, attention, and working memory.

***Capability and wellbeing***

The ICEpop Capability Measure for Adults (ICECAP-A)^18^ measures 5 capabilities (stability, attachment, autonomy, achievement, and enjoyment) that are important to quality of life.

**Table S1.** Full participant characteristics.

| **Characteristics** | **All (n=60)** | **PERFORM (n=40)** | **Control (n=20)** |
| --- | --- | --- | --- |
| **Age, years** | 62.0 (13.0) | 61.4 (12.7) | 63.4 (13.9) |
| **Sex at birth, n (% female)** | 34 (56.7%) | 23 (57.5%) | 11 (55.0%) |
| **Ethnicity, n (%)**  *White British*  *Asian or Asian British*  *Black or Black British*  *Mixed* | 50 (83.3%)  6 (10.0%)  2 (3.3%)  2 (3.3%) | 33 (57.5%)  4 (10.0%)  1 (2.5%)  2 (5.0%) | 17 (85.0%)  2 (10.0%)  1 (5.0%)  0 (0.0%) |
| **BMI, kg/m^2^** | 30.8 (8.0) | 31.2 (8.1) | 30.1 (7.9) |
| **IMD scores** | 6.0 (2.6)  6.5 [4.0, 8.0] | 5.5 (2.7)  6.0 [3.0, 7.0] | 7.2 (2.2)  8.0 [5.5, 9.0] |
| **Highest level of education, n (%)**  *University*  *College*  *Secondary school*  *Other*  *Unknown*  *Missing data* | 16 (26.7%)  18 (30.0%)  13 (21.7%)  1 (1.7%)  2 (3.3%)  10 (16.7%) | 11 (27.5%)  12 (30.0%)  9 (22.5%)  1 (2.5%)  0 (0.0%)  7 (17.5%) | 5 (25.0%)  6 (30.0%)  4 (20.0%)  0 (0.0%)  2 (10.0%)  3 (15.0%) |
| **Employment status, n (%)***  *In full time employment*  *In part time employment*  *Not employed but seeking*  *Not employed, not seeking*  *Retired*  *Unable to work due to ill health*  *Missing data* | 9 (15.0%)  2 (3.3%)  1 (1.7%)  1 (1.7%)  30 (50.0%)  10 (16.7%)  9 (15.0%) | 5 (12.5%)  2 (5.0%)  0 (0.0%)  1 (2.5%)  20 (50.0%)  8 (20.0%)  6 (15.0%) | 4 (20.0%)  0 (0.0%)  1 (5.0%)  0 (0.0%)  10 (50.0%)  2 (10.0%)  3 (15.0%) |
| **Annual household income, n (%)**  *£1 to £9,999*  *£10,000 to £24,999*  *£25,000 to £49,999*  *£50,000 to £74,999*  *£75,000 to £99,999*  *£100,000 or more*  *Declined to answer*  *Missing data* | 6 (10.0%)  14 (23.3%)  12 (20.0%)  4 (6.7%)  1 (1.7%)  1 (1.7%)  10 (16.7%)  12 (20.0%) | 3 (7.5%)  11 (27.5%)  8 (20.0%)  3 (7.5%)  0 (0.0%)  0 (0.0%)  7 (17.5%)  8 (20.0%) | 3 (15.0%)  3 (15.0%)  4 (20.0%)  1 (5.0%)  1 (5.0%)  1 (5.0%)  3 (15.0%)  4 (20.0%) |
| **Marital status, n (%)**  *Married*  *Single*  *Divorced*  *Widowed*  *Cohabiting*  *Prefer not to say*  *Missing data* | 23 (38.3%)  6 (10.0%)  12 (20.0%)  6 (10.0%)  3 (5.0%)  1 (1.7%)  9 (15.0%) | 14 (35.0%)  4 (10.0%)  9 (22.5%)  5 (12.5%)  2 (5.0%)  0 (0.0%)  6 (15.0%) | 9 (45.0%)  2 (10.0%)  3 (15.0%)  1 (5.0%)  1 (5.0%)  1 (5.0%)  3 (15.0%) |
| **Lives alone, n (%)**  *Yes*  *No*  *Prefer not to say*  *Missing data* | 12 (20.0%)  37 (61.7%)  2 (3.3%)  9 (15.0%) | 8 (20.0%)  25 (62.5%)  1 (2.5%)  6 (15.0%) | 4 (20.0%)  12 (60.0%)  1 (5.0%)  3 (15.0%) |
| **Smoking status, n (%)**  *Current*  *Former*  *Never*  *Missing data* | 6 (10.0%)  25 (41.7%)  27 (45.0%)  2 (3.3%) | 4 (10.0%)  19 (47.5%)  16 (40.0%)  1 (2.5%) | 2 (10.0%)  6 (30.0%)  11 (55.0%)  1 (5.0%) |
| **Current vaper, n (%)**  *Yes*  *No*  *Missing data* | 5 (8.3%)  50 (83.3%)  5 (8.3%) | 4 (10.0%)  33 (82.5%)  3 (7.5%) | 1 (5.0%)  17 (85.0%)  2 (10.0%) |
| **Number of LTC, median [IQR]** | 4.0 [3.0, 6.0] | 4.0 [3.0, 6.0] | 4.0 [3.0, 5.5] |
| **Number of LTC with an indication for exercise therapy, median [IQR]** | 3.0 [2.0, 4.0] | 3.0 [2.0, 4.0] | 3.5 [2.0, 4.0] |

*Data are reported as mean (SD), median [IQR], or n (%). BMI, Body Mass Index; IMD, Index of Multiple Deprivation; LTC, long-term conditions. *Some individuals answered yes to more than 1 employment status.*

**Table S2.** Full list of self-reported long-term conditions.

| **Long-term condition** | **All (n=60)** | **PERFORM (n=40)** | **Control (n=20)** |
| --- | --- | --- | --- |
| **Alcohol problems** | 0 (0.0%) | 0 (0.0%) | 0 (0.0%) |
| **Amputation** | 0 (0.0%) | 0 (0.0%) | 0 (0.0%) |
| **Anorexia nervosa or bulimia** | 0 (0.0%) | 0 (0.0%) | 0 (0.0%) |
| **Anxiety** | 19 (31.7%) | 11 (27.5%) | 8 (40.0%) |
| **Arthritis** | 18 (30.0%) | 15 (37.5%) | 3 (15.0%) |
| **Asthma** | 22 (36.7%) | 16 (40.0%) | 6 (30.0%) |
| **Atrial fibrillation** | 6 (6.7%) | 2 (5.0%) | 2 (10.0%) |
| **Bronchiectasis** | 7 (11.7%) | 5 (12.5%) | 2 (10.0%) |
| **Cancer – solid tumour** | 7 (11.7%) | 3 (7.5%) | 4 (20.0%) |
| **Type:**  Localised  Remission  Missing data | 2 (3.3%)  4 (6.7%)  1 (1.7%) | 0 (0.0%)  2 (5.0%)  1 (2.5%) | 2 (10.0%)  2 (10.0.%)  0 (0.0%) |
| **Chronic fatigue syndrome** | 2 (3.3%) | 1 (2.5%) | 1 (5.0%) |
| **Chronic kidney disease** | 14 (23.3%) | 10 (25.0%) | 4 (20.0%) |
| **Chronic liver disease** | 0 (0.0%) | 0 (0.0%) | 0 (0.0%) |
| **Chronic obstructive pulmonary disease** | 12 (20.0%) | 9 (22.5%) | 3 (15.0%) |
| **Chronic sinusitis** | 1 (1.7%) | 0 (0.0%) | 1 (5.0%) |
| **Connective tissue disease** | 0 (0.0%) | 0 (0.0%) | 0 (0.0%) |
| **Coronary heart disease** | 8 (13.3%) | 7 (17.5%) | 1 (5.0%) |
| **Dementia** | 0 (0.0%) | 0 (0.0%) | 0 (0.0%) |
| **Depression** | 16 (26.7%) | 10 (25.0%) | 6 (30.0%) |
| **Diabetes**  **Type:**  Type 1  Type 2 | 25 (41.7%)    9 (15.0%)  16 (26.7%) | 17 (42.5%)    4 (10.0%)  13 (32.5%) | 8 (40.0%)    5 (25.0%)  3 (15.0%) |
| **Diverticular disease** | 2 (3.3%) | 2 (5.0%) | 0 (0.0%) |
| **Endometriosis** | 0 (0.0%) | 0 (0.0%) | 0 (0.0%) |
| **Epilepsy** | 2 (3.3%) | 1 (2.5%) | 1 (5.0%) |
| **Glaucoma** | 1 (1.7%) | 0 (0.0%) | 1 (5.0%) |
| **Heart failure** | 1 (1.7%) | 0 (0.0%) | 1 (5.0%) |
| **Hypertension** | 23 (38.3%) | 14 (35.0%) | 9 (45.0%) |
| **Incontinence (bladder)** | 1 (1.7%) | 0 (0.0%) | 1 (5.0%) |
| **Incontinence (bowel)** | 0 (0.0%) | 0 (0.0%) | 0 (0.0%) |
| **Inflammatory bowel disease** | 2 (3.3%) | 2 (5.0%) | 0 (0.0%) |
| **Irritable bowel syndrome** | 5 (8.3%) | 4 (10.0%) | 1 (5.0%) |
| **Long-COVID** | 11 (18.3%) | 7 (17.5%) | 4 (20.0%) |
| **Meniere's disease** | 0 (0.0%) | 0 (0.0%) | 0 (0.0%) |
| **Migraines** | 5 (8.3%) | 4 (10.0%) | 1 (5.0%) |
| **Multiple sclerosis** | 1 (1.7%) | 0 (0.0%) | 1 (5.0%) |
| **Osteoporosis** | 8 (13.3%) | 6 (15.0%) | 2 (10.0%) |
| **Painful condition** | 21 (35.0%) | 13 (32.5%) | 8 (40.0%) |
| **Parkinson's disease** | 1 (1.7%) | 1 (2.5%) | 0 (0.0%) |
| **Pelvic organ prolapse** | 0 (0.0%) | 0 (0.0%) | 0 (0.0%) |
| **Peripheral vascular disease** | 1 (1.7%) | 1 (2.5%) | 0 (0.0%) |
| **Pernicious anaemia** | 1 (1.7%) | 0 (0.0%) | 1 (5.0%) |
| **Polycystic ovarian syndrome** | 0 (0.0%) | 0 (0.0%) | 0 (0.0%) |
| **Prostate disorders** | 3 (5.0%) | 3 (7.5%) | 0 (0.0%) |
| **Psoriasis or eczema** | 6 (10.0%) | 4 (10.0%) | 2 (10.0%) |
| **Psychoactive substance misuse** | 0 (0.0%) | 0 (0.0%) | 0 (0.0%) |
| **Schizophrenia or bipolar affective disorder** | 0 (0.0%) | 0 (0.0%) | 0 (0.0%) |
| **Stroke or transient ischaemic attack** | 3 (5.0%) | 2 (5.0%) | 1 (5.0%) |
| **Thyroid disease** | 9 (15.0%) | 7 (17.5%) | 2 (10.0%) |
| **Treated constipation** | 4 (6.7%) | 2 (5.0%) | 2 (10.0%) |
| **Treated dyspepsia** | 0 (0.0%) | 0 (0.0%) | 0 (0.0%) |
| **Viral hepatitis** | 0 (0.0%) | 0 (0.0%) | 0 (0.0%) |
| **Other medical condition** | 17 (28.3%) | 11 (27.5%) | 6 (30.0%) |

*Data are reported as frequencies (%).*

**Table S3.** Participant characteristics within each trial site.

| **Characteristics** | **University Hospitals of Leicester (n=20)** | **Newcastle Foundation Hospitals (n=20)** | **Reach for Health (n=20)** |
| --- | --- | --- | --- |
| **Age, years** | 55.7 (12.0) | 69.2 (9.5) | 61.3 (14.0) |
| **Sex at birth, n (% female)** | 11 (55.0%) | 11 (55.0%) | 12 (60.0%) |
| **Ethnicity, n (%)**  *White British*  *Asian or Asian British*  *Black or Black British*  *Mixed* | 11 (55.0%)  5 (25.0%)  2 (10.0%)  2 (10.0%) | 19 (95%)  1 (5.0%)  0 (0.0%)  0 (0.0%) | 20 (100.0%)  0 (0.0%)  0 (0.0%)  0 (0.0%) |
| **BMI, kg/m^2^** | 31.6 (8.6) | 30.3 (6.3) | 30.4 (8.9) |
| **IMD scores** | 5.6 (3.0)  5.0 [3.0, 8.5] | 5.3 (2.7)  6.0 [2.5, 7.0] | 7.3 (1.8)  7.0 [6.0, 8.5] |
| **Highest level of education, n (%)**  *University*  *College*  *Secondary school*  *Other*  *Unknown*  *Missing data* | 10 (50.0%)  8 (40.0%)  2 (10.0%)  0 (0.0%)  0 (0.0%)  0 (0.0%) | 2 (10.0%)  3 (15.0%)  3 (15.0%)  0 (0.0%)  2 (10.0%)  10 (50.0%) | 4 (20.0%)  7 (35.0%)  8 (40.0%)  1 (5.0%)  0 (0.0%)  0 (0.0%) |
| **Employment Status, n (%)***  *Full-time employment*  *Part-time employment*  *Not employed but seeking*  *Not employed, not seeking*  *Retired*  *Unable to work due to ill health*  *Missing data* | 5 (25.0%)  1 (5.0%)  0 (0.0%)  0 (0.0%)  8 (40.0%)  6 (30.0%)  0 (0.0%) | 0 (0.0%)  0 (0.0%)  1 (5.0%)  0 (0.0%)  10 (50.0%)  0 (0.0%)  9 (45.0%) | 4 (20.0%)  1 (5.0%)  0 (0.0%)  1 (5.0%)  12 (60.0%)  4 (20.0%)  0 (0.0%) |
| **Annual household income, n (%)**  *£1 to £9,999*  *£10,000 to £24,999*  *£25,000 to £49,999*  *£50,000 to £74,999*  *£75,000 to £99,999*  *£100,000 or more*  *Declined to answer*  *Missing data* | 1 (5.0%)  7 (35.0%)  6 (30.0%)  2 (10.0%)  1 (5.0%)  0 (0.0%)  1 (5.0%)  2 (10.0) | 0 (0.0%)  1 (5.0%)  0 (0.0%)  1 (5.0%)  0 (0.0%)  0 (0.0%)  9 (45.0%)  9 (45.0%) | 5 (25.0%)  6 (30.0%)  6 (30.0%)  1 (5.0%)  0 (0.0%)  1 (5.0%)  0 (0.0%)  1 (5.0%) |
| **Marital status, n (%)**  *Married*  *Single*  *Divorced*  *Widowed*  *Cohabiting*  *Prefer not to say*  *Missing data* | 7 (35.0%)  5 (25.0%)  5 (25.0%)  2 (10.0%)  1 (5.0%)  0 (0.0%)  0 (0.0%) | 6 (30.0%)  0 (0.0%)  2 (10.0%)  1 (5.0%)  1 (5.0%)  1 (5.0%)  9 (45.0%) | 10 (50.0%)  1 (5.0%)  5 (25.0%)  3 (15.0%)  1 (5.0%)  0 (0.0%)  0 (0.0%) |
| **Lives alone**  *Yes*  *No*  *Prefer not to say*  *Missing data* | 4 (20.0%)  16 (80.0%)  0 (0.0%)  0 (0.0%) | 4 (20.0%)  7 (35.0%)  0 (0.0%)  9 (45.0%) | 4 (20.0%)  14 (70.0%)  2 (10.0%)  0 (0.0%) |
| **Smoking status, n (%)**  *Current smoker*  *Ex-smoker*  *Never smoked*  *Missing data* | 2 (10.0%)  7 (35.0%)  11 (55.0%)  0 (0.0%) | 1 (5.0%)  10 (50.0%)  8 (40.0%)  1 (5.0%) | 3 (15.0%)  8 (40.0%)  8 (40.0%)  1 (5.0%) |
| **Current vaper**  *Yes*  *No*  *Missing data* | 1 (5.0%)  19 (95.0%)  0 (0.0%) | 1 (5.0%)  18 (90.0%)  1 (5.0%) | 3 (15.0%)  13 (65.0%)  4 (20.0%) |
| **Number of LTC, median [IQR]** | 4.0 [3.0, 5.0] | 4.5 [4.0, 6.0] | 5.0 [2.5, 6.0] |
| **Number of LTC with an indication for exercise therapy, median [IQR** | 3.3 (1.1)  3.0 [2.0, 4.0] | 3.8 (1.3)  4.0 [3.0, 4.5] | 3.0 (1.2)  3.0 [2.0, 4.0] |

**Risk of bias details**

Overall, there were 7 outcome assessor breaks. This was due to 6 follow-up assessments being conducted by the same assessor as the baseline assessments at one site, and one unblinding over the phone at another.

**Table S4.** Fried’s frailty results.

|  | **PERFORM** | | **Control** | |
| --- | --- | --- | --- | --- |
|  | **Baseline** | **3-month follow-up** | **Baseline** | **3-month follow-up** |
| **Physical frailty, n (%):** |  |  |  |  |
| Robust | 8 (20.0%) | 8 (20.0%) | 2 (10.0%) | 2 (10.0%) |
| Intermediate | 7 (17.5%) | 12 (30.0%) | 9 (45.0%) | 6 (30.0%) |
| Frail | 2 (5.0%) | 1 (2.5%) | 0 (0.0%) | 0 (0.0%) |
| N, Missing assessment for one or more criteria | 23 | 19 | 9 | 12 |
| **Slowness (Low gait speed), n (%)** | 7 (21.9%) | 9 (28.1%) | 2 (12.5%) | 4 (25.0%) |
| **Weakness (Low grip strength), n (%)** | 11 (31.4%) | 10 (34.5%) | 6 (35.3%) | 6 (50.0%) |
| **Exhaustion, n (%)** | 8 (25.0%) | 9 (27.3%) | 6 (40.0%) | 5 (35.7%) |
| **Unintentional weight loss, n (%)** | 1 (3.3%) | 6 (18.1%) | 0 (0.0%) | 0 (0.0%) |
| **Low physical activity, n (%)** | 7 (25.0%) | 5 (17.2%) | 3 (21.4%) | 5 (50.0%) |

*Frail if >=3 criteria present; Intermediate or Pre-Frail if 1 or 2 criteria present; Robust if 0 criteria present. For each individual criteria the percentage shown is calculated based only on those with data for that variable while the percentage for physical frailty classifications are calculated from all participants.

**Table S5.** Incremental shuttle walk test secondary outcome results.

|  | **PERFORM (n=40)** | | | **Control (n =20)** | | | **Between-group difference of the change (95% CI)** |
| --- | --- | --- | --- | --- | --- | --- | --- |
|  | **Baseline** | **3-month follow-up** | **Mean change (95% CI)** | **Baseline** | **3-month follow-up** | **Mean change (95% CI)** |  |
| **Post-ISWT measures** | | | | | | | |
| **HR, bpm** | 92 (20) | 97 (17) | 1 (-7, 9) | 104 (20) | 102 (32) | 3 (-14, 20) | -2 (-17, 14) |
| *Missing data* | 14 | 9 | 20 | 8 | 7 | 10 | 30 |
| **SpO2, %** | 95 (4) | 95 (4) | 0 (-2, 2) | 96 (3) | 96 (2) | 0 (-2, 2) | 0 (-4, 3) |
| *Missing data* | 14 | 9 | 20 | 8 | 6 | 10 | 30 |
| **Systolic blood pressure** | 149 (19) | 154 (28) | 4 (-6, 13) | 172 (42) | 166 (36) | 12 (-4, 28) | -8 (-25, 8) |
| *Missing data* | 15 | 9 | 21 | 8 | 8 | 11 | 32 |
| **Diastolic blood pressure** | 80 (10) | 83 (14) | 2 (-4, 8) | 90 (17) | 90 (16) | 7 (-12, 26) | -6 (-20, 9) |
| *Missing data* | 15 | 9 | 21 | 8 | 8 | 11 | 32 |
| **BORG breath** | 3.5 (1.9) | 3.8 (1.9) | 0.9 (-0.2, 2.0) | 3.6 (0.9) | 4.8 (2.7) | 1.8 (-0.3, 3.9) | -0.9 (-2.9, 1.1) |
| *Missing data* | 15 | 8 | 20 | 8 | 6 | 10 | 30 |
| **BORG exertion** | 5.0 (3,1) | 4.6 (2.3) | 0.3 (-1.4, 1.9) | 5.4 (2.8) | 4.9 (2.1) | -0.1 (-2.5, 2.3) | 0.4 (-2.4, 3.1) |
| *Missing data* | 14 | 8 | 20 | 8 | 6 | 10 | 30 |
| **Reason for termination**  **N recorded as terminating**  *Shortness of breath, n (%)*  *Leg fatigue, n (%)*  *Pain, n (%)*  *Other, n (%)* | **26 (100%)**  7 (26.9%)  3 (11.5%)  9 (34.6%)  13 (50.0%) | **32 (100%)**  13 (40.6%)  7 (21.9%)  10 (31.3%)  11 (34.4%) |  | **12 (100%)**  3 (25.0%)  1 (8.3%)  4 (33.3%)  5 (41.7%) | **13 (100%)**  8 (61.5%)  3 (23.1%)  4 (30.8%)  4 (30.8%) |  |  |
| *Missing data* |  |  |  |  |  |  |  |

N.B. several individuals recorded more than one reason for termination and therefore the rows total more than 100% for this measure.

**Table S6.** Mean fidelity score for each item for the health and wellbeing sessions.

| **Item** | **Mean score + standard deviation** |
| --- | --- |
| Person centred communication | 2.9 + 0.9 |
| Supporting action planning | 0.9 + 1.0 |
| Review progress | 2.1 + 1.0 |
| Support and encourage problem solving | 1.1 + 1.1 |
| Use of ask-tell-discuss | 2.6 + 1.1 |
| Keep discussion focused | 3.7 + 1.0 |
| Normalise psychological consequences | 1.7 + 1.1 |
| Engage social support | 0.8 + 1.0 |
| Signpost to relevant resources | 1.1 + 1.5 |

**Figure S1.** Bar chart showing the quantity of each healthcare resource category used between baseline and three months across all study participants.

**
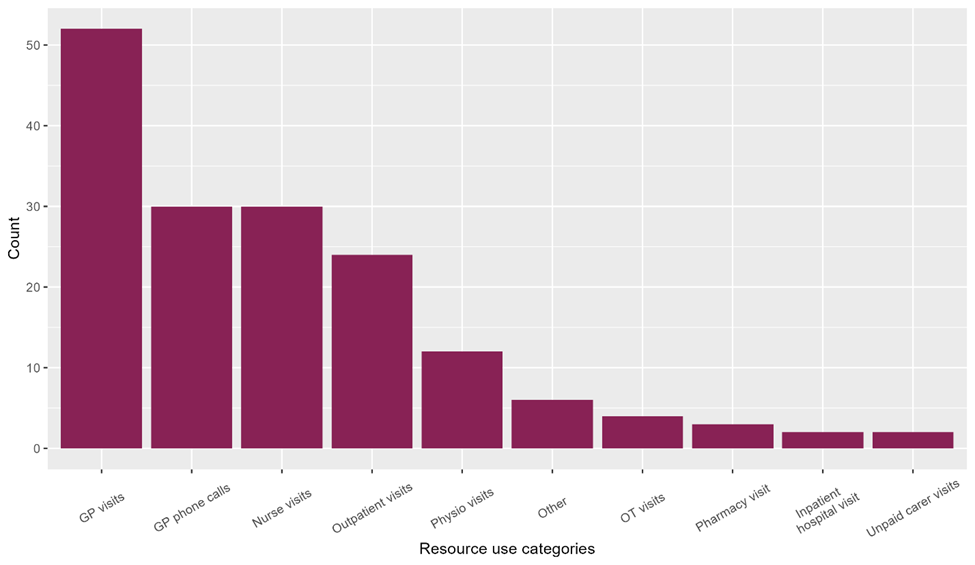
**

*Other resource use included dentist visits, chiropractor and talking therapy through Improving Access to Psychological Therapies.*

**Figure S2.** EQ-5D-5L Domain scores at baseline and 3 months.


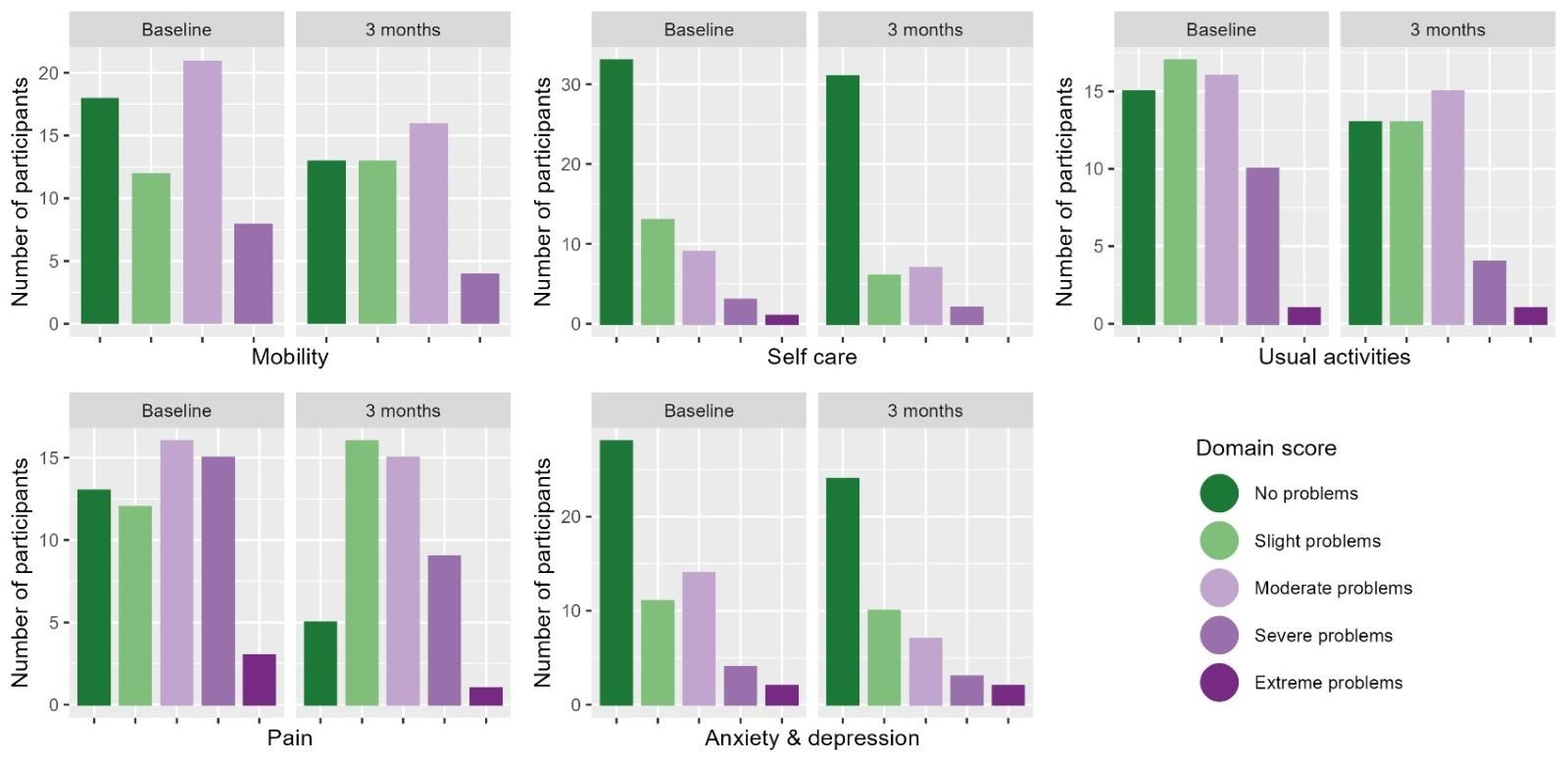


**Figure S3.** ICECAP-A Domain scores at baseline and 3 months follow-up.


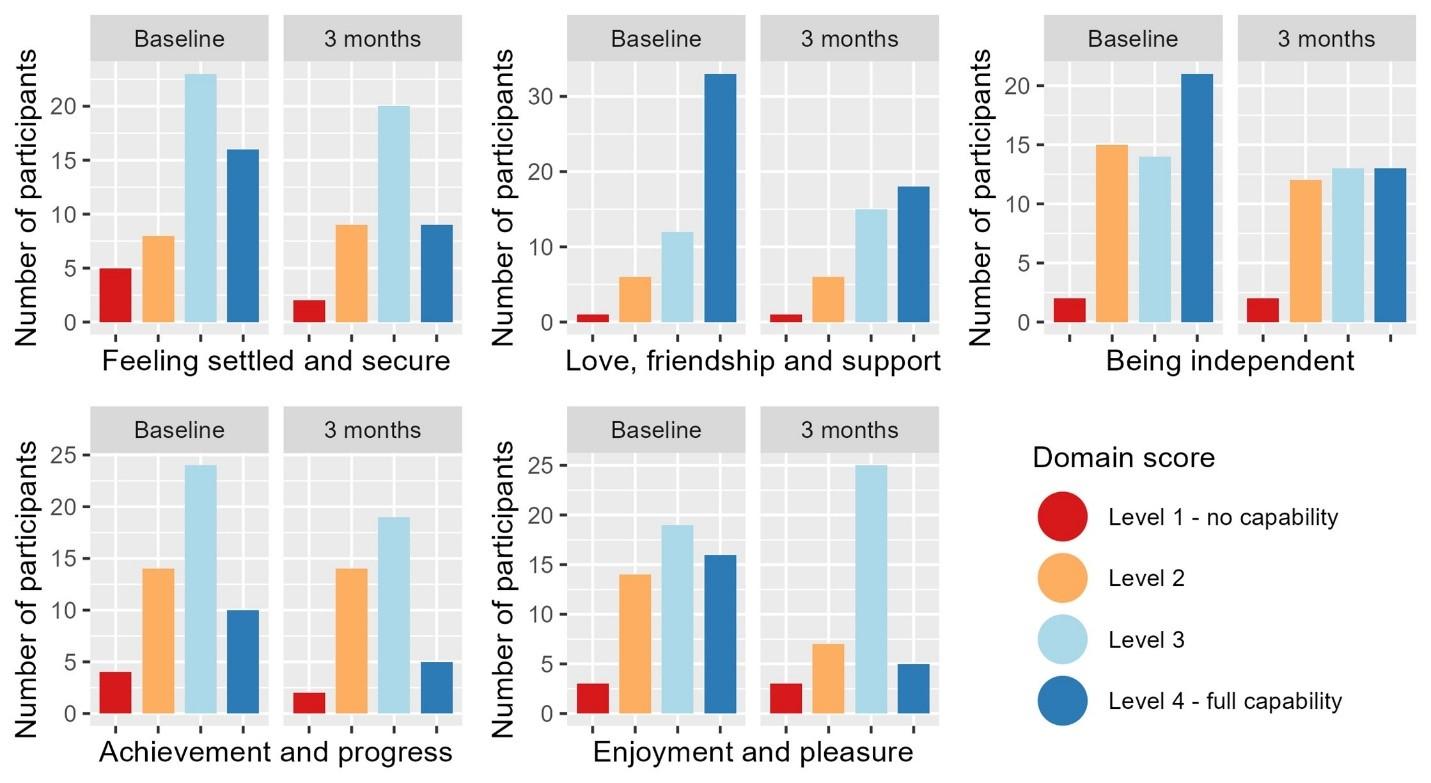


**REFERENCES**

1. Singh SJ, Morgan M, Scott S, Walters D, Hardman AE. Development of a shuttle walking test of disability in patients with chronic airways obstruction. *Thorax*. 1992;47(12):1019-1024.

2. Kon SS, Patel MS, Canavan JL, et al. Reliability and validity of 4-metre gait speed in COPD. *European Respiratory Journal*. 2013;42(2):333-340.

3. Leong DP, Teo KK, Rangarajan S, et al. Prognostic value of grip strength: findings from the Prospective Urban Rural Epidemiology (PURE) study. *The lancet*. 2015;386(9990):266-273.

4. Herdman M, Gudex C, Lloyd A, et al. Development and preliminary testing of the new five-level version of EQ-5D (EQ-5D-5L). *Quality of life research*. 2011;20:1727-1736.

5. Nolan CM, Longworth L, Lord J, et al. The EQ-5D-5L health status questionnaire in COPD: validity, responsiveness and minimum important difference. *Thorax*. 2016;71(6):493-500.

6. Kroenke K, Spitzer RL, Williams JB. The PHQ‐9: validity of a brief depression severity measure. *Journal of general internal medicine*. 2001;16(9):606-613.

7. Spitzer RL, Kroenke K, Williams JB, Löwe B. A brief measure for assessing generalized anxiety disorder: the GAD-7. *Archives of internal medicine*. 2006;166(10):1092-1097.

8. Webster K, Cella D, Yost K. The F unctional A ssessment of C hronic I llness T herapy (FACIT) Measurement System: properties, applications, and interpretation. *Health and quality of life outcomes*. 2003;1:1-7.

9. Cleeland CS, Ryan K. The brief pain inventory. *Pain Research Group*. 1991;20(20):143-7.

10. Yorke J, Moosavi SH, Shuldham C, Jones PW. Quantification of dyspnoea using descriptors: development and initial testing of the Dyspnoea-12. *Thorax*. 2010;65(1):21-26.

11. Craig CL, Marshall AL, Sjöström M, et al. International physical activity questionnaire: 12-country reliability and validity. *Medicine & science in sports & exercise*. 2003;35(8):1381-1395.

12. Stewart AL, Ware JE. *Measuring functioning and well-being: the medical outcomes study approach*. duke university Press; 1992.

13. Duncan P, Murphy M, Man M-S, Chaplin K, Gaunt D, Salisbury C. Development and validation of the multimorbidity treatment burden questionnaire (MTBQ). *BMJ open*. 2020;8(4):e019413.

14. Fried LP, Tangen CM, Walston J, et al. Frailty in older adults: evidence for a phenotype. *The Journals of Gerontology Series A: Biological Sciences and Medical Sciences*. 2001;56(3):M146-M157.

15. Carleton RN, Thibodeau MA, Teale MJ, et al. The center for epidemiologic studies depression scale: a review with a theoretical and empirical examination of item content and factor structure. *PloS one*. 2013;8(3):e58067.

16. Organisation WH. WHO Disability Assessment Schedule 2.0 (WHODAS 2.0). Accessed December 2024, <https://www.who.int/standards/classifications/>

17. Nasreddine ZS, Phillips NA, Bédirian V, et al. The Montreal Cognitive Assessment, MoCA: a brief screening tool for mild cognitive impairment. *Journal of the American Geriatrics Society*. 2005;53(4):695-699.

18. Al-Janabi H, N Flynn T, Coast J. Development of a self-report measure of capability wellbeing for adults: the ICECAP-A. *Quality of life research*. 2012;21:167-176.
