# Supplementary material for: Personalised exercise-rehabilitation for people with multiple long-term conditions (PERFORM): a randomised feasibility study
Source: BMJ Open. 2025 Sep 17;15(9):e100195. doi: 10.1136/bmjopen-2025-100195 (PMC12458827; doi:10.1136/bmjopen-2025-100195)
Supplement: Supplementary file 2 [file bmjopen-2025-100195.reviewer_comments.pdf]

PEER REVIEW HISTORY

BMJ Open publishes all reviews undertaken for accepted manuscripts. Reviewers are asked to complete a checklist review form and are provided with free text boxes to elaborate on their assessment. These free text comments are reproduced below.

ARTICLE DETAILS

Title (Provisional)

Personalised Exercise-Rehabilitation FOR people with Multiple long-term conditions (PERFORM): a randomised feasibility study

Authors

Evans , Rachael A; Simpson, Sharon Anne Anne; Manifield, James R; Ahmed, Zahira; Barber, Shaun; Barwell, Gwen; Brown, Sophie Eleanor; Daw, Paulina; Dean, Sarah Gerard; Doherty, Patrick Joseph; Fraser, Heather; Gardiner, Nikki; Greaves, Colin; Ibbotson, Tracy; Jani, Bhautesh; Jolly, Kate; Mair, Frances; McIntosh, Emma; Megaritis, Dimitrios; Miller, Daniel; Ormandy, Paula; Smith, Susan; Vogiatzis, Ioannis; Waheed, Ghazala; Withers, Tom M; Taylor, Rod; Singh, Sally J; research team, PERFORM

VERSION 1 - REVIEW

**Reviewer**	**1**
**Name**	**Stokes, Tim**
**Affiliation**	**Otago University, General Practice & Rural Health**
**Date**	**09-Apr-2025**
**COI**	**None**

This paper reports a feasibility RCT study of PERFORM - a personalised exercise-rehabilitation programme for people with multimorbidity.

This is a well designed and conducted feasibility study and the introduction clearly sets out the current gaps in the evidence base that support the proposed RCT in this area.

The findings support progression to a full RCT and it is noted that the study importantly identifies a number of adaptations - presented in box 1 - that are needed for the design of the full RCT. Further, the limitations section clearly identifies the rationale for a 'centre-based" modality which I consider is justified for this specific RCT design.

**Reviewer**	**2**
**Name**	**Saco-Ledo, Gonzalo**

Affiliation **Universidad Europea de Madrid Campus de Villaviciosa de Odón, INVESTIGACION Y DOCTORADO**

Date **19-May-2025**

COI **None**

The study entitled "Personalised Exercise-Rehabilitation FOR people with Multiple long-term conditions (PERFORM): a randomised feasibility study" focuses on assessing the feasibility and acceptability of a newly developed personalised exercise-rehabilitation programme for people with multiple long-term conditions (MLTC). This is a highly relevant and timely topic, particularly for a journal such as BMJ, and in my view, the study has good potential for acceptance following major revision. I would like to offer a few comments that I believe could help the authors improve the manuscript:

- Sample Size Justification: It is important to clarify the rationale behind the chosen sample size. Why were 60 participants included in the study? Was a formal sample size calculation conducted? If so, I recommend including it in the manuscript. Additionally, please justify the 2:1 randomization ratio. Providing this information will help readers better understand the study design and its methodological rigor.

- Strengths and Limitations Section: I suggest separating the strengths and limitations of the study into two distinct paragraphs to improve readability. Furthermore, the limitations section should be strengthened. For example, consider addressing the small sample size, the specific characteristics of the participant population, and issues related to missing data, among other relevant points. These additions will provide a more balanced and transparent assessment of the study’s limitations.

VERSION 1 - AUTHOR RESPONSE

Reviewer: 1

Prof. Tim Stokes, Otago University

Comments to the Author:

This paper reports a feasibility RCT study of PERFORM - a personalised exercise-rehabilitation programme for people with multimorbidity.

C1This is a well designed and conducted feasibility study and the introduction clearly sets out the current gaps in the evidence base that support the proposed RCT in this area.

The findings support progression to a full RCT and it is noted that the study importantly

identifies a number of adaptations - presented in box 1 - that are needed for the design of the full RCT. Further, the limitations section clearly identifies the rationale for a 'centre-based" modality which I consider is justified for this specific RCT design.

R1 Many thanks for your review and constructive comments.

Reviewer: 2

The study entitled "Personalised Exercise-Rehabilitation FOR people with Multiple long-term conditions (PERFORM): a randomised feasibility study" focuses on assessing the feasibility and acceptability of a newly developed personalised exercise-rehabilitation programme for people with multiple long-term conditions (MLTC). This is a highly relevant and timely topic, particularly for a journal such as BMJ, and in my view, the study has good potential for acceptance following major revision. I would like to offer a few comments that I believe could help the authors improve the manuscript:

C1 Sample Size Justification: It is important to clarify the rationale behind the chosen sample size. Why were 60 participants included in the study? Was a formal sample size calculation conducted? If so, I recommend including it in the manuscript. Additionally, please justify the 2:1 randomization ratio. Providing this information will help readers better understand the study design and its methodological rigor.

R1 We have now further clarified our rationale for the sample size within the randomisation section of the methods and cited the following guidelines (Lancaster, G. A., Dodd, S., & Williamson, P. R. (2004). Design and analysis of pilot studies: recommendations for good practice. *Journal of evaluation in clinical practice*, *10*(2), 307-312.):

"We aimed to recruit 60 participants to allow us to assess feasibility of the intervention and trial methods (Lancaster et al., 2004)". "Forty participants within the intervention group allowed for at least two cohorts of PERFORM per site with 6-7 participants in each. Furthermore, the 2:1 randomisation ratio maximised recruitment to the intervention group whilst allowing for an understanding of feasibility under randomised conditions."

C2 Strengths and Limitations Section: I suggest separating the strengths and limitations of the study into two distinct paragraphs to improve readability. Furthermore, the limitations section should be strengthened. For example, consider addressing the small sample size, the specific characteristics of the participant population, and issues related to missing data, among other relevant points. These additions will provide a more balanced and transparent assessment of the study's limitations.

R2 Thank you for your suggestion. We have now separated the strengths and limitations section into two paragraphs and added the following to the limitations:

"The relatively small sample size and level of data completeness within this feasibility trial should be noted. Our future multicentre RCT aims to be fully powered to a priori detect significant changes in the primary outcome (EQ-5D-5L)."
